# Dissecting non-B DNA structural motifs in untranslated regions of eukaryotic genomes

**DOI:** 10.1186/s44342-024-00028-x

**Published:** 2024-11-27

**Authors:** Aruna Sesha Chandrika Gummadi, Divya Kumari Muppa, Venakata Rajesh Yella

**Affiliations:** Department of Biotechnology, Koneru Lakshmaiah Education Foundation, Vaddeswaram, Guntur, Andhra Pradesh 522302 India

**Keywords:** Untranslated region, Non-B DNA, Curved DNA, G-quadruplex, Z-DNA

## Abstract

The untranslated regions (UTRs) of genes significantly impact various biological processes, including transcription, posttranscriptional control, mRNA stability, localization, and translation efficiency. In functional areas of genomes, non-B DNA structures such as cruciform, curved, triplex, G-quadruplex, and Z-DNA structures are common and have an impact on cellular physiology. Although the role of these structures in cis-regulatory regions such as promoters is well established in eukaryotic genomes, their prevalence within UTRs across different eukaryotic classes has not been extensively documented. Our study investigated the prevalence of various non-B DNA motifs within the 5′ and 3′ UTRs across diverse eukaryotic species. Our comparative analysis encompassed the 5′-UTRs and 3′UTRs of 360 species representing diverse eukaryotic domains of life, including Arthropoda (Diptera, Hemiptera, and Hymenoptera), Chordata (Artiodactyla, Carnivora, Galliformes, Passeriformes, Primates, Rodentia, Squamata, Testudines), Magnoliophyta (Brassicales), Fabales (Poales), and Nematoda (Rhabditida), on the basis of datasets derived from the UTRdb. We observed that species belonging to taxonomic orders such as Rhabditida, Diptera, Brassicales, and Hemiptera present a prevalence of curved DNA motifs in their UTRs, whereas orders such as Testudines, Galliformes, and Rodentia present a preponderance of G-quadruplexes in both UTRs. The distribution of motifs is conserved across different taxonomic classes, although species-specific variations in motif preferences were also observed. Our research unequivocally illuminates the prevalence and potential functional implications of non-B DNA motifs, offering invaluable insights into the evolutionary and biological significance of these structures.

**Supplementary Information:**

The online version contains supplementary material available at 10.1186/s44342-024-00028-x.

## Introduction

Untranslated regions (UTRs) are non-coding segments that are located at both the 5′ and 3′ ends of coding sequences in mRNAs and have a significant regulatory impact on gene expression [1]. UTRs play key roles in multifarious levels of gene regulation, including transcription mechanisms, mRNA stability, and translation and its efficiency, even though they do not encode proteins [2, 3]. The crucial gatekeepers that start the translation process are the 5′ UTRs. Their unique secondary structures, upstream open reading frames (uORFs), and sequence motifs control how well the ribosome binds to mRNA, which affects translation efficiency. 3′ UTRs, which are equally important as 5′ UTRs and serve as essential hubs for posttranscriptional regulation, are also significant. RBPs (RNA-binding proteins) and miRNAs (microRNAs) bind to these regions and are critical in controlling the translational stability and efficiency of mRNAs. The 3′ UTRs also contain regulatory elements that, through their interactions with transcription factors and critical roles in the recruitment of RNA-binding proteins and microRNAs, affect the transcription of mRNAs and subsequent posttranscriptional modifications [4]. The length and structural complexity of UTRs are correlated with the sensitivity of gene dosage as well as the regulatory needs of a specific gene [1, 5]. Furthermore, dysregulation of gene expression caused by mutations and alterations in UTR sequences can contribute to several diseases, including cancer [5]. Notably, the majority of studies on UTRs have focused mainly on their role in modulating translation through their structural properties. However, at the level of DNA, the sequence composition and the DNA structure of UTRs can influence the transcription process and efficiency.

Under normal conditions, B-DNA represents the most energetically favorable structure of DNA, requiring energy to alter its conformation. Owing to the interactions between various proteins and DNA during transcription and replication, supercoiling spontaneously occurs within living organisms and serves as a major source of energy required for alterations in DNA structure [6]. Cruciform DNA, curved DNA, hairpins, G-quadruplexes, slipped DNA, triplexes, and Z-DNA are examples of unique non-B DNA structures that form in vivo as a result of specific sequence arrangements combined with the B-DNA super helicity state and play a role in multifarious cellular functions [7]. Curved DNA, which is formed mainly by unique A-tracts [8], can either help or hinder transcription factors and the transcription machinery from binding to them by affecting the accessibility of DNA regions to transcription-related proteins. Furthermore, curved DNA can modify the topological state of the DNA helix, thereby mitigating or intensifying torsional stress during transcription elongation, and curved DNA can also influence DNA supercoiling. Triplex DNA, a third strand of DNA that attaches to the major groove of the double helix to form triplex DNA, also known as H-DNA, is a triple-stranded structure [1]. By preventing RNA polymerase from moving freely along the DNA template, this motif can prevent transcription elongation. DNA structures known as cruciforms are produced by palindromic sequences that fold back on themselves to create cross-shaped patterns. Cruciforms can act as physical barriers during transcription elongation, impeding the progression of RNA polymerase [9]. G-quadruplexes are composed of guanine-rich sequences that stack into G-tetrads, which form four-stranded DNA structures and Hoogsteen hydrogen bonds that stabilize this stacking. These patterns can be found in promoter regions, telomeres, and other regulatory regions. By obstructing RNA polymerase and influencing transcription elongation, G-quadruplexes can control transcription [10–12]. They also affect DNA supercoiling by maintaining particular DNA helix topological states. Slipped DNAs are looped-out regions resulting from repetitive sequences misaligning during DNA replication or transcription, and advancement may be hampered by these structures [13]. Z-DNA formation is linked to particular sequences, such as tracts that alternate between purines and pyrimidines. As a regulatory element, this motif affects transcription by influencing transcription factor binding and transcription initiation. Z-DNA is a left-handed helical form of DNA that can be induced by negative supercoiling and is characterized by its zigzag backbone [7, 10, 14]. The non-canonical B DNA structures in living cells or in vitro can be identified via various approaches, such as spectroscopy, electrophoresis, crystallization, immunology, or sequencing [15]. However, for high-throughput analysis at the genome scale, a well-defined pattern search via computational approaches is preferred. Many studies have utilized computational methods to predict non-B DNA structures in whole genomes, promoter regions [16], and origins of replication across various life domains [16, 17]. However, their prevalence in different eukaryotic forms of life, particularly in untranslated regions (UTRs), has not been comprehensively reported. In our study, we aimed to assess the prevalence of non-B DNA-forming sequences in 360 eukaryotic species belonging to 16 taxonomic orders to report their similarities and differences.

### Datasets

We compiled untranslated region (UTR) data from UTRdb [18] (http://utrdb.cloud.ba.infn.it/utrdb/download.html) for our comprehensive analysis. Our dataset encompasses a total of 360 species (Table 1, Supplementary Table S1), with 3301843 3′-UTRs and 4005237 5′ UTRs, covering species diversity across various phyla, classes, and orders. Notably, there is significant diversity within the insect order Arthropoda (Insecta), with 39 species in the Diptera order, 8 in the Hemiptera order, and 13 in the Hymenoptera order. Among the avian class Aves in the phylum Chordata, there are 6 species in Galliformes and 17 in Passeriformes. The diversity of Mammalia in Chordata is evident, with 16 species in Artiodactyla, 19 in Carnivora, 24 in Primates, and 22 in Rodentia. In the Reptilia group of Chordata, there are 7 species each in the Squamata and Testudines. The phylum Magnoliophyta shows considerable plant diversity in Liliopsida, with 25 species in Poales, and in Magnoliopsida, with 6 species each in Brassicales and Fabales. Nematoda (Secernentea) is diverse among nematodes, with seven species in the order Rhabditida. Furthermore, we considered 138 species falling under miscellaneous categories with orders having fewer than 5 species.

**Table 1:** Datasets used in this study

S.No	Phylum	Class	Order	Species
1	Arthropoda	Insecta	Diptera	39
2	Arthropoda	Insecta	Hemiptera	8
3	Arthropoda	Insecta	Hymenoptera	13
4	Chordata	Aves	Galliformes	6
5	Chordata	Aves	Passeriformes	17
6	Chordata	Mammalia	Artiodactyla	16
7	Chordata	Mammalia	Carnivora	19
8	Chordata	Mammalia	Primates	24
9	Chordata	Mammalia	Rodentia	22
10	Chordata	Reptilia	Squamata	7
11	Chordata	Reptilia	Testudines	7
12	Magnoliophyta	Liliopsida	Poales	25
13	Magnoliophyta	Magnoliopsida	Brassicales	6
14	Magnoliophyta	Magnoliopsida	Fabales	6
15	Nematoda	Secernentea	Rhabditida	7
16	Other	Other	Other	138
Total number of species				360

We collected the UTR information of 360 species from the UTRdb and classified them into 16 phylogenetic groups on the basis of NCBI taxonomy

### Data processing

For our analysis of untranslated region (UTR) data, we employed regular expression models to detect six potential non-B DNA-forming sequences, namely, curved DNA (APR), slipped DNA, G-quadruplexes (GQs), cruciform DNA, triplex DNA, and Z-DNA [19]. (I) Curved DNA, or A-phased repeats, is characterized by the presence of three or more adenine tracts (A-tracts), each containing 3 to 5 adenines spaced exactly 10 nucleotides apart. This arrangement causes a pronounced curvature in the DNA helix. The origin of curved DNA motifs lies in the rigidity of A-tracts. These sequences resist bending in the normal direction of the B-DNA helix, instead inducing a smooth curvature. This deviation often occurs in regulatory regions such as promoters where DNA bending facilitates the binding of transcription factors and other regulatory proteins. (2) Slipped DNA is recognized by the presence of direct repeats (DRs), which are typically 10–50 nucleotides long. These repeats are identical sequences that appear consecutively with no intervening nucleotides, leading to potential misalignments during replication. Slipped DNA structures arise from replication slippage, a process where repetitive sequences misalign, causing loops or bulges in the DNA strand. This phenomenon is common in areas with repetitive sequences and can result in genomic instability or expansion of microsatellites, contributing to genetic disorders. (3) G-quadruplexes are formed by guanine-rich sequences, consisting of four or more runs of G-tracts (3–5 guanines per tract), separated by spacers of 1–7 nucleotides. The guanine bases form planar structures known as G-tetrads through Hoogsteen hydrogen bonding. G-quadruplex formation is driven by the unique ability of guanine to form hydrogen-bonded G-tetrads. These structures are stabilized by cations (potassium or sodium) and often occur in telomeric regions, promoters, or other regulatory zones, where they play a role in stabilizing the DNA and influencing processes such as transcription and replication. (4) Cruciform DNA structures are identified by the presence of inverted repeats (IRs), typically 10–100 nucleotides long, separated by a small spacer of 0–3 nucleotides. The inverted repeats allow the DNA to fold back on itself, forming hairpin or cruciform structures. Cruciform structures originate from sequences with palindromic symmetry. They tend to form under conditions of negative supercoiling, where the torsional stress on the DNA prompts it to adopt alternative conformations. Cruciform DNA is often found at recombination hotspots and can serve as a site for genetic rearrangement or regulation. (5) Triplex DNA is identified by mirror repeats (MRs) of 10–100 nucleotides separated by spacers of 0–8 nucleotides. These motifs are predominantly composed of purine or pyrimidine bases (at least 90% purine or pyrimidine). Triplex DNA originates from sequences that allow the binding of a third DNA strand into the major groove of a duplex, forming a triple-stranded structure. This formation is favored in regions rich in purines and pyrimidines and is often associated with regulatory elements where triplex structures can modulate gene expression. (6) Z-DNA is identified by alternating runs of G-Y sequences (where G is guanine and Y is either cytosine or thymine), typically extending over 10 nucleotides or more. This configuration promotes a left-handed helical structure. Z-DNA forms in regions with alternating purine‒pyrimidine sequences and is stabilized by torsional strain from negative supercoiling during transcription. This unusual left-handed form of DNA is transient and is often linked to the regulation of gene expression during active transcription. Further, we have also evaluated the prevalence of short tandem repeats (STRs). STRs also known as microsatellites, consist of repeating units of 1 to 6 nucleotides. These sequences can occur in both coding and non-coding regions and vary in length between different individuals. STRs originate from the inherent instability of short repetitive sequences, which are prone to slippage during DNA replication. This process results in the expansion or contraction of the repeat number, contributing to genetic diversity and, in some cases, to diseases such as Huntington’s disease or Fragile X syndrome.

Our primary objective was to interpret patterns in these non-B DNA sequences found in the UTRs. To this end, we used the non-B DNA motif association of the UTR, as reported in our previous work [16]. The non-B DNA motif association of a species refers to the percentage of untranslated regions (UTRs) within the species genome that contain at least one instance of a specified non-B DNA sequence motif. This metric is calculated by identifying the presence of these motifs in the entire UTR dataset for the species. A higher percentage suggests a greater prevalence of non-B DNA motifs in a given species, which could indicate evolutionary conservation or functional significance in the regulation of gene expression. For brevity, we use motif associations throughout the manuscript.

## Results

The main goal of this study was to explore the prevalence of non-B DNA in the untranslated regions (UTRs) of a wide range of living organisms. To achieve this goal, we collected genome sequences from 360 organisms (Supplementary Table S1) spanning 16 taxonomic groups (Table 1, Datasets). In this study, we establish links between different patterns within UTRs and their associations with the GC content and average UTR length in each genome. We also report the general features of non-B DNA in UTRs across different taxonomic categories, highlighting noteworthy species.

### Analysis of the relationships among non-B DNA motifs, the GC content, and UTR length

We conducted an extensive analysis of the GC composition and average length of the 5′ and 3′ UTRs across various taxonomic classes. Our findings revealed substantial variation in both average lengths and percentages of GCs. For example, the average 5′-UTR length across all groups was approximately 241.5 nucleotides (± 161.0), whereas the mean length for 3′-UTRs was 639.05 nucleotides (± 340.07). Remarkable extremes were observed, with the nematode *Pristionchus pacificus* displaying the shortest mean 5′-UTR length of 35.7 nucleotides, whereas the yellow fever mosquito *Aedes albopictus* presented the longest length of 1653.9 nucleotides. Similarly, the Asian Swamp Eel *Monopterus albus* had the longest mean 3′-UTR at 1987.2 nucleotides, whereas the Lyre-leaved Rockcress *Arabidopsis lyrata* had the shortest 3′-UTR at 73.0 nucleotides. The median percentage of the 5′-UTR was 48.7% (± 9.9), and the median percentage of the 3′-UTR was 39.5% (± 6.9). Notably, the parasitic nematode *Strongyloides ratti* had the lowest GC percentage at 18.6%, while the alga *Ostreococcus lucimarinus* had the highest at 73.0%. The pineapple *Ananas comosus* presented the highest GC percentage in the 3′-UTRs at 51.7%, whereas that of *Strongyloides ratti* was the lowest at 15.6% (Supplementary Table S1). Significant differences were also noted among the taxonomic orders. For example, in the Arthropoda phylum, substantial differences among Insecta orders, such as Diptera, Hymenoptera, and Hemiptera, have been observed. Diptera had mean 5′-UTR and 3′-UTR lengths of 244.9 and 534.9 nucleotides, respectively, with GC% values of 40.1 and 35.0. Hymenoptera presented mean lengths of 277.3 nucleotides (GC% = 39.6) for 5′-UTRs and 635.2 nucleotides (GC% = 29.2) for 3′-UTRs. Conversely, Hemiptera had shorter UTRs, with mean lengths of 207.9 nucleotides (GC% = 37.7) for 5′-UTRs and 342.2 nucleotides (GC% = 28.2) for 3′-UTRs. In conclusion, our analysis highlights the substantial variability in UTR length and GC content across different taxonomic groups, shedding light on their diverse regulatory mechanisms and evolutionary adaptations.

**Fig. 1:**
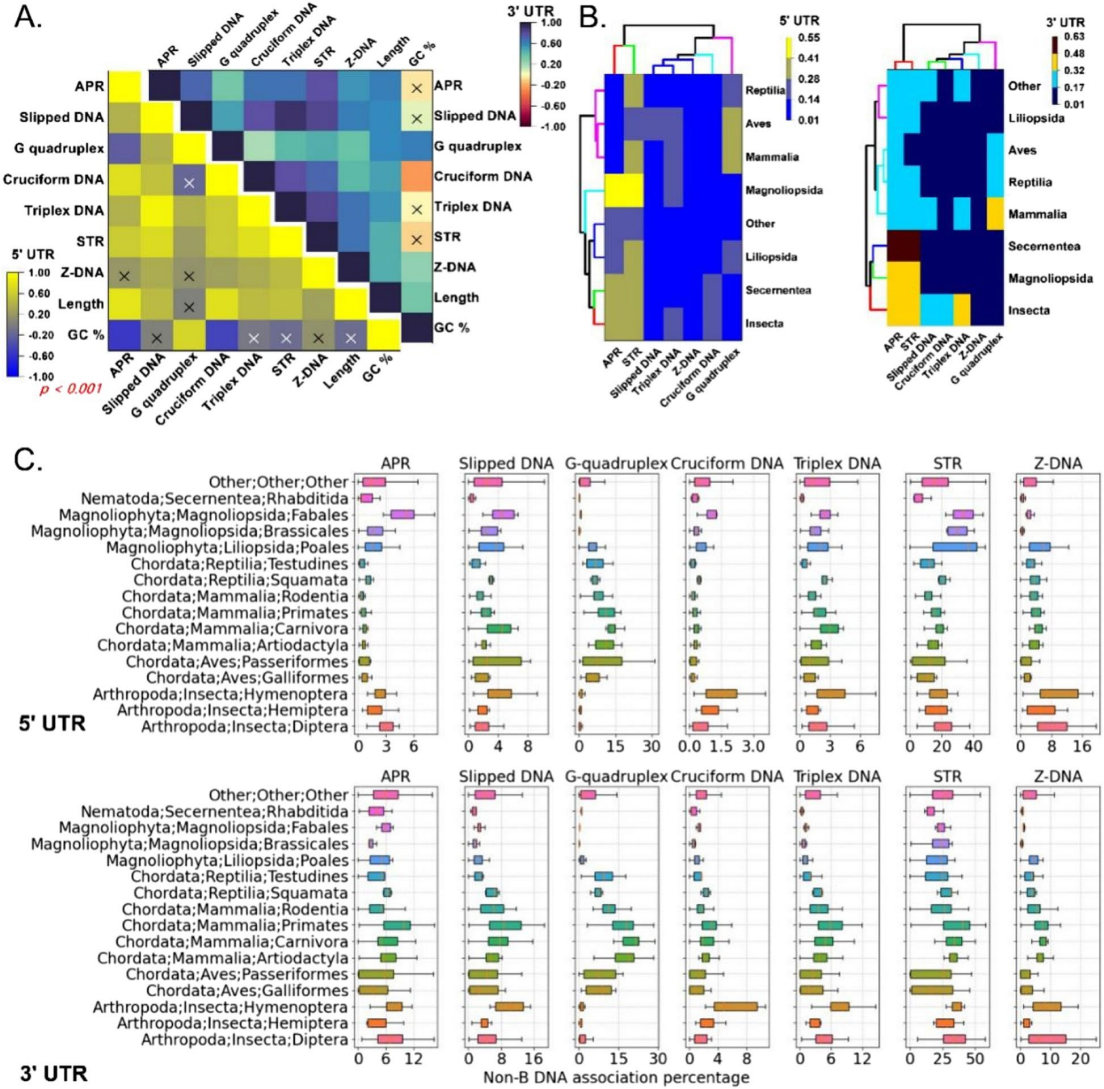
Analysis of non-B DNA structures across different UTR regions and taxonomic groups. **A** Correlation heatmap depicting the correlation between different non-B-DNA structures in the 3′UTR and 5′UTR. The “X” values in the plot indicate that correlation values are not significant (*p* = 0.001). **B** Hierarchical clustering of taxonomic groups on the basis of non-B-DNA structures in the 3′UTR and 5′UTR. **C** Boxplots of association percentages of different non-B-DNA structures in different taxonomic groups

We then performed an analysis to investigate the connections between length, GC content, and different non-B DNA motifs in 360 species, with a particular emphasis on the 5′ and 3′ UTRs independently. Significant relationships (Pearson correlation coefficient, *p* < 0.001) between different motifs, GC content, and UTR length within the 5′ and 3′ UTRs were observed (Fig. 1A). Strong correlations between G-quadruplex structures (0.6358 in 3′ UTRs and 0.66912 in 5′ UTRs) and the GC content were found, suggesting that these structures have the propensity to form in GC-rich regions. Conversely, we found inverse correlations of the GC content with curved DNA motifs (APRs) and cruciform DNA (− 0.52302 and − 0.44116 in 5′ UTRs, respectively), highlighting a distinct pattern distribution influenced by the nucleotide composition. Additionally, UTR length showed strong correlations with multiple motifs, including APR (0.79021 in 5′ UTRs and 0.63775 in 3′ UTRs) and cruciform DNA (0.74699 in 5′ UTRs), and moderate correlations with other motifs, such as triplex DNA and STR, suggesting that longer UTRs can accommodate a broader range of regulatory elements. We also detected significant cooccurrences between motifs, as indicated by the strong correlation between triplex DNA and slipped DNA (0.9171 in 3′ UTRs), likely arising from shared functional necessities in gene regulation.

To understand the distribution of non-B DNA across taxonomic classes, we standardized the non-B DNA association percentage with the mean UTR length of each species within their respective taxonomic classes for the 5′ and 3′ UTRs. We then used the Euclidean distance and average linkage methods to construct cluster diagrams (Fig. 1B). The analysis revealed three principal clusters for non-B DNA associations in different taxonomic classes: (i) curved DNA and STR prevalence; (ii) G-quadruplex prevalence; and (iii) slipped DNA, cruciform DNA, triplex DNA, and Z-DNA distribution (Fig. 1B). We present these results in subsequent sections, with a focus on taxonomic classes and some important species.

### Curved DNA and STR motifs are predominant in insects, nematodes, and flowering plants

The occurrence of curved DNA motifs, known as A-phased repeats (APRs), is high in Insecta, Secernentea (nematodes), and Magnoliopsida (flowering plants) (Fig. 1C, Supplementary Table S2). These motifs are abundant in both the 5′ and 3′ untranslated regions (UTRs). Secernentea shows elevated levels of APR in the 3′ UTRs, indicating unique regulatory adaptations. The frequency of APR motifs varies significantly across different orders within UTR regions. For example, Diptera (insect) has a median APR association of 2.96% in the 5′ UTRs, whereas Primates present the highest median APR association of 9.77% in the 3′ UTRs. Among the 3′ (5.07%) and 5′ (0.31%) UTRs, testudines have the lowest median APR value at 5′ UTRs, and 3′ Hemiptera has the lowest median APR value. Additionally, APR associations have been noted for particular species, such as *Folsomia candida* (White Springtail), *Oryzias latipes* (Japanese Medaka), and *Theobroma cacao* (Cacao Tree), with 15.90%, 27.5%, and 12.5%, respectively. These results indicate that APR motifs are uniquely enriched and might play a role in regulatory mechanisms. Notably, short tandem repeats (STRs) were also enriched in the majority of the taxonomic orders enriched with APR motifs, indicating their co-occurrence. Almonds (*Prunus dulcis*) presented the strongest STR association (48.79%) in the 5′ UTR, whereas the 3′ UTR of Japanese medaka (*Oryzias latipes*) presented an astounding 66.99% association, emphasizing the regulatory significance of STRs. Similarly, peach (*Prunus persica*) was associated with 47.64% of the 5′ UTR, and sorghum (*Sorghum bicolor*) was associated with 47.12% of the 5′ UTR and 3′ UTR. Furthermore, the leech (*Helobdella robusta*) and aphid wasps (*Aphidius gifuensis*) presented 45.53% and 53.79% STR associations in the 5′ UTR and 3′ UTR, respectively, demonstrating the prevalence of STRs in aquatic invertebrates and parasitoid insects.

### G-quadruplexes are predominant in mammals, reptiles, and Aves

**Fig. 2:**
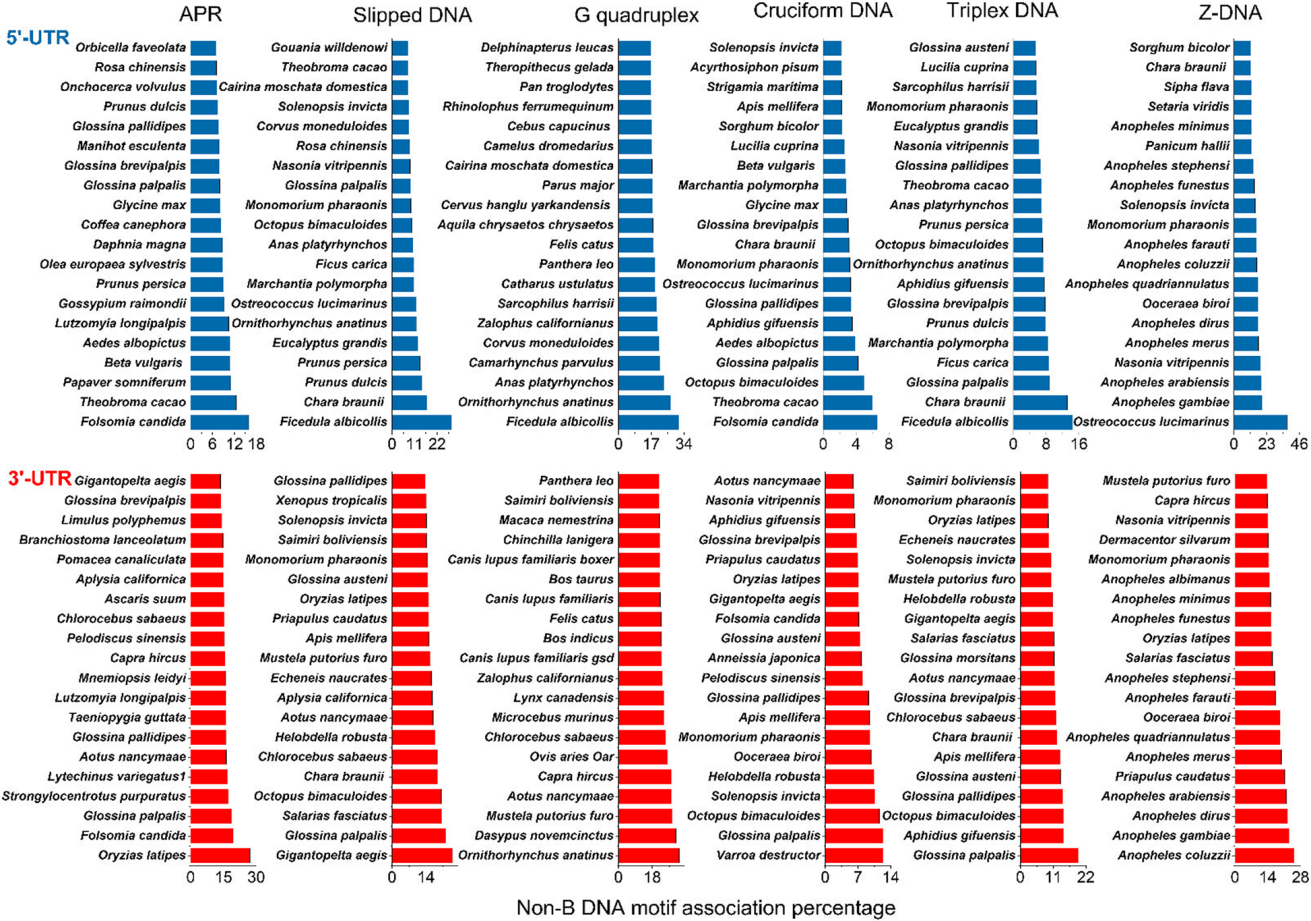
Supplementary Table S3. The top 20 organisms with the highest frequency of non-BDNA motif repeats in both the 3′UTR and 5′UTR. Here, blue represents the organisms that have the greatest number of non-B DNA motifs in the 5′UTR, and red represents the organisms that have the greatest number of non-B DNA motifs in the 3′UTR

G-quadruplexes (GQs) are extremely stable structures made of guanine-rich sequences that are essential for maintaining telomeres, controlling gene expression, and maintaining genomic stability [7, 11, 13, 20]. The significant correlation that our analysis revealed between GQs in different species suggests the evolutionary significance of these traits. With noticeably high median association values, G-quadruplex occurrence varies greatly among taxa (Fig. 2, Supplementary Table S3). For example, in the 5′ UTRs, Carnivora within Mammalia had the highest median association at 13.70%, whereas Rhabditida within Nematoda presented the lowest median association at 0.07% (Fig. 2, Supplementary Table S3, Supplementary Table S1). In the 3′ UTRs, Carnivora also had a high median association of 21.46%, whereas Brassicales within Magnoliophyta presented the lowest median association at 0.04%. The mean association values for Carnivora in the 5′ UTRs (12.58%) and 3′ UTRs (18.52%), with CVs of 0.42 and 0.40, indicate consistent motif prevalence. Conversely, Rhabditida’s low mean values (5′ UTRs: 0.09%, 3′ UTRs: 0.93%) and high CVs (5′ UTRs 1.16, 3′ UTRs 0.97) reflect sparse and variable occurrences. *Ficedula albicollis* (collared flycatcher), an avian species from the Phylum Chordata, Class Aves, Order Passeriformes, presented the highest association of GQs in the 5 UTR at 31.14%, highlighting the potential regulatory complexity within avian genomes. The high frequencies of GQs suggest a complex level of gene regulation and genomic stability in birds. In the 3′ UTR, *Ornithorhynchus anatinus* (Platypus) has a GQ of 32.38%, indicating the important role of GQs in mammalian genomic regulation, particularly in unique monotreme species [12, 21].

G-quadruplexes (GQs) are essential for maintaining telomeres, regulating genes, and maintaining genomic stability in a variety of species [22]. A 23.70% GQ association was detected in the 5′ UTR of the mallard duck, indicating the importance of GQs in regulating genes related to avian development and adaptation. Small Tree Finch was also found to have a GQ association of 21.38%, indicating the importance of this motif in regulating bird genomes. Nancy Ma’s Night Monkey, a primate, showed a GQ association of 57.95% in the 3′ UTR, indicating the regulatory significance of this motif in monkeys. Moreover, the California sea lion presented a GQ association of 20.13% in the 5′ UTR, suggesting the regulatory significance of this motif in marine vertebrates. The domestic goats presented a 54.06% GQ association in the 3′ UTR, demonstrating the motif’s role in regulating genes essential for domesticated animals.

### Different taxonomic classes have distinct distributions of slipped DNA, cruciform DNA, triplex DNA, and Z-DNA

The variety of slipped DNA motifs found in various taxa greatly contributes to genomic stability [13] (Fig. 2, Supplementary Table S3). Testudines have the lowest median association value (0. 99%) among the 5′ UTRs, whereas Hymenoptera had the highest value (3.51%). With respect to the 3′ UTRs, Hymenoptera had the highest median association value (10.24%), whereas Rhabditida had the lowest value (1.28%). These discoveries provide insight into the regulatory mechanisms of these motifs and their connection to disorders marked by genomic instability and repeat expansion [23, 24]. The highest percentage of slipped DNA motifs was found in the 5′ UTR (29.05%) with the Collared Flycatcher and in the 3′ UTR (24.92%) with the deep-sea snail *Gigantopelta aegis*. In the 5′ UTR, almond and peach displayed associations of 14.63% and 13.64%, respectively. In the 3-unit UTR, aphid wasps showed a 14.52% association. The 3′ (4.91%) and 5′ (1.66%) UTRs presented high median association values, indicating the presence of cruciform DNA motifs in Hymenoptera. The 3′ (1.70%) and 5′ (0.15%) UTRs indicate that Rodentia has the lowest median values. Within the 3′ UTR, Varroa Mite demonstrated a 12.35% association, whereas Tsetse Fly demonstrated a 14.27% association.

High median association values were observed for the Hymenoptera species for the 3′ (8.17%) and 5′ (3.05%) UTR triplex DNA motifs. On the other hand, in the 3′ (0.48%) and 5′ (0.21%) UTRs, Rhabditida had the lowest median association values. Hymenoptera (5′ UTRs 3.40%, 3′ UTRs 8.07%) and Rhabditida (5′ UTRs 0.64%, 3′ UTRs 0.43%) are known to exhibit consistent triplex motifs; however, the lower mean values in Rhabditida suggest low occurrence. Triplex DNA motifs are present in many different species and are important in the regulation of genes. Certain species exhibit a high frequency of triplex DNA associations [25, 26]. These include the collared flycatcher *Ficedula albicollis*, which has a 14.45% association in the 5′ UTR; the Japanese Medaka *Oryzias latipes*, which has a 15.53% association in the 3′ UTR; the chlorophyte alga *Chara braunii*, which has a 13.14% association in the 5′ UTR; and the aphid *Aphidius gifuensis*, which has an 11.49% association in the 3′ UTR. Furthermore, the mammals with significant associations in the 5′ and 3′ UTRs included *Ornithorhynchus anatinus* (Platypus) and *Octopus bimaculoides* (California two-spot Octopus). Z-DNA motifs differ significantly: Brassicales within Magnoliophyta have low median association values in both the 5′ (0.47%) and 3′ UTRs (0.60%), whereas Carnivora has high median association values in both the 5′ (4.20%) and 3′ UTRs (7.83%). Z-DNA motifs are highly and consistently present in Carnivora (5′ UTRs 4.53%, 3′ UTRs 7.27%) and Brassicales (5′ UTRs 0.43%, 3′ UTRs 0.38%), although they are rare in Brassicales. Because these are left-handed helical structures, Z-DNA is required for the stability and expression of these motifs in the genome [10, 27]. For example, with a 37.20% association, *Ostreococcus lucimarinus* (Marine Green Alga) had the highest Z-DNA occurrence in the 5′ UTR. *Glossina austeni*, the tsetse fly, displayed a 51.41% association in the 3′ UTR. In the 3′ UTR, *Anopheles gambiae* was associated with 23.13% Z-DNA, whereas the malaria mosquito *Anopheles coluzzii* was associated with 25.30% Z-DNA. *Aedes albopictus* showed a 10.84% association with the 5′ UTR. *Salarias fasciatus* (Jeweled Blenny) showed a 15.95% association in the 5′ UTR, whereas *Priapulus caudatus* (Penis Worm) showed a 21.23% association in the 3′ UTR. Z-DNA motifs are widely distributed, which emphasizes their importance.

## Discussion

Untranslated regions play key roles in cellular homeostasis through the modulation of gene expression [21, 28, 29]. The 5′ UTR can affect transcription initiation by modifying the binding of transcription factors and RNA polymerase. Certain sequences in the 5′ UTR can either enhance or silence transcriptional activity [3]. Elements such as polyadenylation signals are found in the 3′ UTR and are essential for appropriate transcription termination. These sequences control the machinery responsible for cleavage and polyadenylation, ensuring that transcription ends at the appropriate time and that the mRNA is processed correctly [30].

While previous studies have focused primarily on promoter regions, cis-regulatory regions, or genomic scales, we have reported the functional importance of non-B DNA structures and different types of non-B DNA structures, such as curved DNA, slipped DNA, G-quadruplexes, cruciform DNA, triplex DNA, short tandem repeats (STRs), and Z-DNA in untranslated regions (UTRs), across 360 species to provide a comprehensive understanding of the evolutionary and functional implications of these motifs. Untranslated regions (UTRs) play important roles in gene regulation by interacting with non-B DNA structures and modulating DNA supercoiling. These interactions influence transcription initiation, elongation, and termination. Non-B DNA structures, such as G-quadruplexes in the 5′ UTR, can either enhance or hinder the assembly of the transcription machinery, affecting transcription initiation. Similarly, triplex DNA and cruciforms can slow down RNA polymerase during elongation, impacting transcription efficiency. At termination sites, Z-DNA can induce local supercoiling, influencing transcriptional termination and contributing to transcript diversity. UTRs regulate translation efficiency and mRNA stability. In the 5′ UTR, non-B DNA structures can control ribosome binding, affecting how much protein is synthesized. The 3′ UTR, on the other hand, influences mRNA decay and stability by interacting with RNA-binding proteins and miRNAs. UTRs also contain signals for alternative polyadenylation, leading to mRNA isoforms with varied stability and localization. These processes are essential for posttranscriptional regulation, ensuring proper gene expression in different cellular contexts. UTRs contribute to genome evolution and stability. By harboring non-B DNA structures such as short tandem repeats or G-quadruplexes, UTRs can drive genetic variation and evolution. This variability allows organisms to adapt to environmental changes but can also lead to genetic disorders when mutations occur. Thus, UTRs, through their interactions with non-B DNA structures, are crucial for the fine-tuning of gene regulation and play a significant role in development, cellular function, and evolutionary adaptation.

However, the non-B DNA motif prevalence in the eukaryotic domain of life has been found to exhibit both similarities and differences among taxonomic groups in our current study [5, 21, 28]. The lengths and GC contents of the UTRs varied significantly among the taxonomic groups we observed. This variation reflects the different evolutionary pressures and functional requirements of these regions in different organisms. R-loops, composed of RNA‒DNA hybrids and displaced single-stranded DNA, can form in UTRs and influence transcription initiation. R-loops can cause transcriptional pausing and facilitate the formation of non-B DNA structures such as Z-DNA and G-quadruplexes, impacting the accessibility of the transcriptional machinery [31]. We aimed to gain insights into the functional significance of these motifs through a comparative study. Our recent study, which focused on the cis-regulatory regions of 1180 genomes, demonstrated that non-B DNA structures are evolutionarily constrained. Additionally, two other independent studies revealed the evolutionary expansion of non-canonical DNA sequences in several eukaryotes. Experimental findings have shown that the composition of G-quadruplexes in mammals is linked to evolutionary divergence time. Importantly, although non-B DNA structures are associated with genome instability, mutations, and diseases, they are under positive purifying selection during evolution.

Recent studies have revealed that distinct curved DNA structures are present in a range of taxonomic groups, suggesting a variety of regulatory roles. These curved DNA motifs are essential for regulating DNA supercoiling and transcription factor accessibility. According to our research, high levels of APRs are observed in Diptera (a class of Arthropoda) and Primates (a class of Mammalia), suggesting that these animals play a major role in genomic regulation. Moreover, our study revealed that the taxonomic orders with lower APR levels are Hemiptera and Testudines, and these orders might have different regulatory mechanisms. While there are certain similarities between the distinct non-B DNA patterns in the 5′ and 3′ UTRs, the organisms have different frequencies and regulatory roles. For example, the highest levels of APRs are observed in the 5′ UTRs of arthropod species such as Diptera, which may help in the binding of initiation factors and ribosome assembly to improve translation initiation. APRs have the highest frequency in the 3′UTR in primates belonging to the order Mammalia, indicating their significance in terms of mRNA stability and posttranscriptional localization. These differences suggest that the structural characteristics and regulatory roles of curved DNA may be influenced by evolutionary pressures, which indirectly affect the frequency and distribution of curved DNA among various taxa.

G-quadruplexes, or GQs, are frequently observed in various functional regions and are important for gene expression regulation, genomic stability, and telomere preservation. These structures are very important because they are conserved even through evolution and have a high affinity for RNA-binding proteins. Our research revealed species-specific differences, indicating that GQs are present only in specific taxa and suggesting various regulatory mechanisms. G-quadruplexes in the 5′ or 3′ UTR can act as physical barriers to transcription elongation. These four-stranded structures, formed by guanine-rich sequences, can cause RNA polymerase to pause or terminate prematurely, impacting the elongation process [32, 33].

Furthermore, slipped DNA motifs are linked with neurodegenerative diseases involving genomic stability and repeat expansion in human genomes [24] and are also enriched in Rhabditida (Nematoda) and Hymenoptera (Insecta). In the 3′UTR of Hymenoptera, cruciform DNA is found, which may be linked to the binding of regulatory proteins that affect mRNA stability and localization. These findings suggest that the accessibility of the transcription machinery can be controlled by cruciform DNA. In contrast, our research revealed the presence of cruciform and triplex DNA (H-DNA) motifs in a wide range of taxonomic groups, suggesting their significant role in gene regulation. Finally, the frequency of Z-DNA motifs in specific taxonomic orders highlights their importance in regulating gene expression and maintaining genomic stability, underscoring their adaptations specific to individual species. The prevalence of motifs such as slipped DNA and triplex DNA in these regions is noteworthy. The higher frequency of slipped DNA motifs in Hymenoptera in 5′ UTR Insecta may be associated with maintaining genomic stability prior to the beginning of transcription. By obstructing the binding of RNA polymerase and termination factors, triplex DNA structures in the 3′ UTR can function as regulatory elements and modulate transcription termination [34]. The greater frequency of triplex DNA motifs in the 3′ UTR of Hymenoptera aids in understanding the potential regulatory role of this gene in mRNA processing and stability during posttranscriptional elongation. Z-DNA formation results in torsional strain, which affects transcription elongation. This strain can enhance or inhibit the progression of RNA polymerase activity along the DNA template. Z-DNA is associated with transcriptionally active regions, suggesting its role in modulating elongation efficiency [35]. The different patterns of non-B DNA motif presence in the 5′ and 3′ UTRs may reveal their specific roles in transcription elongation. It is well known that non-B DNA structures can alter transcription. This research further develops upon previous studies by conducting a thorough analysis of non-B DNA motifs in a range of eukaryotic species.

## Supplementary Information

Supplementary Material 1: Supplementary Table S1: Datasets used in this study. We collected genome sequences corresponding to 5'-UTR and 3'-UTR for 360 organisms from UTRdb (http://utrdb.cloud.ba.infn.it/utrdb/download.htm). NCBI taxonomy (https://www.ncbi.nlm.nih.gov/taxonomy) is used to classify species into different taxonomic groups namely Phylum, Class and Order. Table S2: The median values of all the orders. Table S3: Top 20 organisms which has highest frequency of Non-BDNA motifs repeats in them in both 3'-UTR and 5'-UTR.

## Data Availability

The generated data is submitted as supplementary table. Raw downloaded datasets will be avialable from corresponding author from request.
